# Impact of perioperative decreased serum albumin level on anastomotic leakage in esophageal squamous cell carcinoma patients treated with neoadjuvant chemotherapy followed by minimally invasive esophagectomy

**DOI:** 10.1186/s12885-023-11713-5

**Published:** 2023-12-08

**Authors:** Ying-Jian Wang, Xian-Feng Xie, Yi-Qiu He, Tao Bao, Xian-Dong He, Kun-Kun Li, Wei Guo

**Affiliations:** 1 Department of Thoracic Surgery, Army Medical Center of PLA (Daping Hospital), Changjiang Route #10, Daping, Chongqing, 400042 PR China; 2 Department of pediatrics, Shapingba District Maternity & Infant Health Hospital, Tiancheng Route #2, Shapingba, Choingqing, 401331 PR China

**Keywords:** Esophageal cancer, Serum albumin, Anastomotic leakage, Minimally invasive esophagectomy, Neoadjuvant chemotherapy

## Abstract

**Background:**

Anastomotic leakage (AL) is a severe complication following esophagectomy with high mortality. Perioperative decreased serum albumin level is considered a predictive of AL, however, its impact on AL incidence in patients treated with neoadjuvant chemotherapy (NCT) followed by minimally invasive esophagectomy (MIE) is not well defined.

**Methods:**

The data of 318 consecutive esophageal cancer patients who underwent MIE were collected retrospectively from January 2021 to December 2021. The perioperative level of albumin was detected and the baseline of altering levels for albumin was established. The incidence of postoperative complications and survival rate were analyzed between groups.

**Results:**

After exclusion, 137 patients were enrolled and assigned to more decreased albumin (MA) and less decreased albumin (LA) groups. The levels of albumin descended significantly after MIE (*p* < 0.0001). There was no significant difference in the clinicopathologic characteristics or surgical outcomes between groups. The incidence of postoperative AL was 10.2% in MA group and 1.4% in LA group (*p* = 0.033). Three patients died due to AL in MA group, while no mortality was observed in LA group (*p* = 0.120). The rate of other postoperative complications was similar between groups. Progression-free survival (PFS) in LA group was a little higher than that in MA group, but it was no significant difference (*p* = 0.853). Similarly, no difference was observed in overall survival (OS) between groups (*p* = 0.277).

**Conclusions:**

Severely deficient serum albumin after MIE was an indicator of AL in esophageal cancer patients treated with NCT.

**Trial registration:**

Chinese clinical trial registry: ChiCTR2200066694, registered December14th,2022. https://www.chictr.org.cn/edit.aspx?pid=185067&htm=4.

## Introduction

The surgical resection is the cornerstone for the treatment of esophageal cancer with curative intention. Even though giant leaps in perioperative management and evolved surgical techniques are contributed to a decreased trend of occurrence of postoperative complications, anastomotic leakage (AL) remains the one of most severe complications after esophagectomy with a high incidence ranging from 11.4% to 21.2% [1–4]. Notably, for the development of AL, surgical technique, neoadjuvant therapy, nutritional status, comorbidities and anatomic location are considered as the most critical parameters [5]. A plethora of risk factors have been identified as the possible reason for AL development but do not come forward with sufficient evidence to determine the optimal treatment strategy. Therefore, the identification of reliable and decisive factors is essential for prevention and treatment of AL after surgery.

To date, with the wide acceptance of the concept of multidisciplinary modality in cancer treatment, neoadjuvant therapy plays an important role in the treatment of esophageal neoplasm [6–8]. However, neoadjuvant therapy may carry the risk of multi-organ dysfunction by the main cause of toxicity, e.g., anemia and hepatic dysfunction. As a major clinical spectrum associated with adverse events after neoadjuvant therapy, as a common manifestation, malnutrition was observed in many esophageal cancer patients. Meanwhile, the level of serum albumin, which are sensitive indicators for both nutrition status and tissue healing, was associated with the occurrence of AL [9]. Moreover, as modifiable parameters, serum albumin may guide an optimal patient-tailored management of perioperative strategy. However, whether the decreased perioperative serum albumin level has the impact on esophageal cancer patients treated with neoadjuvant therapy followed by surgery is still controversial. Therefore, the aim of this study is to investigate the relationship between decreased perioperative serum albumin level and AL in esophageal squamous cell cancer (ESCC) patients who underwent neoadjuvant therapy followed by minimally invasive esophagectomy (MIE) and to guide a patient-tailored optimization of treatment strategy.

## Methods

### Study design

This study was a single-center, retrospective, observational study (ChiCTR2200066694) to evaluate the impact of perioperative decreased perioperative serum albumin level on AL after neoadjuvant chemotherapy (NCT) followed by MIE in ESCC patients. This study was conducted in accordance with the Good Clinical Practice guidelines and the Declaration of Helsinki and approved by the Ethics Committee of the Army Medical Center of PLA (Daping Hospital) (number:2,022,300). Because this was a retrospective study, written informed consent was not required. This study is reported according to the Strengthening the Reporting of Observational Studies in Epidemiology (STROBE) guidelines.

### Evaluation of serum albumin

The levels of serum albumin were measured by blood samples preoperatively and for seven consecutive days after surgery. The lowest level of postoperative serum albumin was collected for analysis. After comparing the preoperative and lowest postoperative level of serum albumin, the baseline of the average decreased levels (percentage) was determined. The arithmetic mean perioperative decreased levels of serum albumin (26.3%) and were defined as cutoff for grouping.

### Patient eligibility and grouping

The study was conducted by chart review of 318 consecutive patients who underwent esophagectomy between January 2021 and December 2021 in the thoracic department of Army Medical Center of PLA (Daping Hosptital). To eliminate the heterogeneity of the study, only ESSC patients who received NCT followed by MIE were included. The main inclusion criteria were: (a) ESCC patients; (b) treated with NCT; (c) McKeown MIE with curative intention; (d) available data of perioperative serum ALB; (e) without early AL (occurred within 48 h after surgery).

After exclusion, patients were divided into two groups separately according to the perioperative decreased serum albumin level. Group MA (more decreased albumin) contained the patients whose decreased serum ALB level was more than the average, and Group LA (less decreased albumin) included those whose decreased serum albumin level was less than the average.

### Neoadjuvant chemotherapy

Two cycles of chemotherapy were administered preoperatively. The regimen was continuously intravenous drip infusion paclitaxel at a dose of 135 mg/m2 based on body surface area over 4 h on day 1 and cisplatin at a dose of 75 mg/m2 based on body surface area over 4 h on day 2. Hydration was given intravenously before, during and after cisplatin infusion. All patients received prophylactic anti-nausea treatment during chemotherapy. The interval between the two cycles was 3 weeks.

### Surgery

Patients were scheduled to undergo surgery 4–6 weeks after preoperative treatment. All patients underwent a three-stage MIE (McKeown procedure), including thoracoscopic esophageal mobilization, laparoscopic gastric mobilization and lymph node dissection. If the cervical lymph nodes were found to be enlarged in contrast-enhanced computed tomography (CT) preoperatively, then a three-field lymphadenectomy was performed. Otherwise, only a two-field lymphadenectomy was performed. A cervical esophagogastric anastomosis was created after the above procedure was completed. The details of the surgical procedure have been described previously in another study [9].

### Study outcomes

Intraoperative data, postoperative complications and pathological parameters were obtained by chart review. The Clavien–Dindo system was used to evaluate the severity of postoperative complications.

### AL definition and diagnosis

AL was defined as full thickness of gastroesophageal tract defect involving esophagus, anastomosis, staple lines, or conduit irrespective of presentation or method of identification according to *International Consensus on Standardization of Data Collection for Complications Associated With Esophagectomy* [10].

The anastomosis was checked consistently every day after surgery. AL was diagnosed by the presentation of enteric content, endoscopic visualization of a defect in the anastomosis, or by extravasation of radiography with water-soluble contrast medium radiography or computed tomography.

### Endpoints

The primary endpoint was the occurrence of AL. Secondary endpoints included progression-free survival (PFS; the time from the start of neoadjuvant therapy to tumor progression), disease-specific survival (DSS; the time from the start of neoadjuvant therapy to death from a specific disease), and postoperative complications. The postoperative pathologic stage was assessed according to the 8th edition of the American Joint Committee on Cancer / Union for International Cancer Control (AJCC/UICC) staging system [11].

### Follow-up

Patients were followed up every 3 months during the first 2 years, every 6 months for the following 3 years, and then annually after 5 years. The duration of follow-up was calculated from the date of the first cycle of neoadjuvant therapy to the date of the last contact or death. The final follow-up was performed on 15th, September 2022.

### Statistics

Values are expressed as the mean ± standard deviation. Continuous data were compared using the Mann–Whitney U test, and categorical data of the two groups were compared by Fisher’s exact test. Pearson correlation analysis was used for detecting the relationship between postoperative serum albumin. The survival rate was calculated using the Kaplan–Meier method, and a log-rank test was used to assess the survival differences between groups. The *p* values < 0.05 were considered statistically significant. Statistical analyses were performed using MedCalc 12 (MedCalc Software, Ostend, Belgium) and GraphPad Prism 5 (GraphPad Software, CA, USA).

## Results

### Patient characteristics

**Fig. 1:**
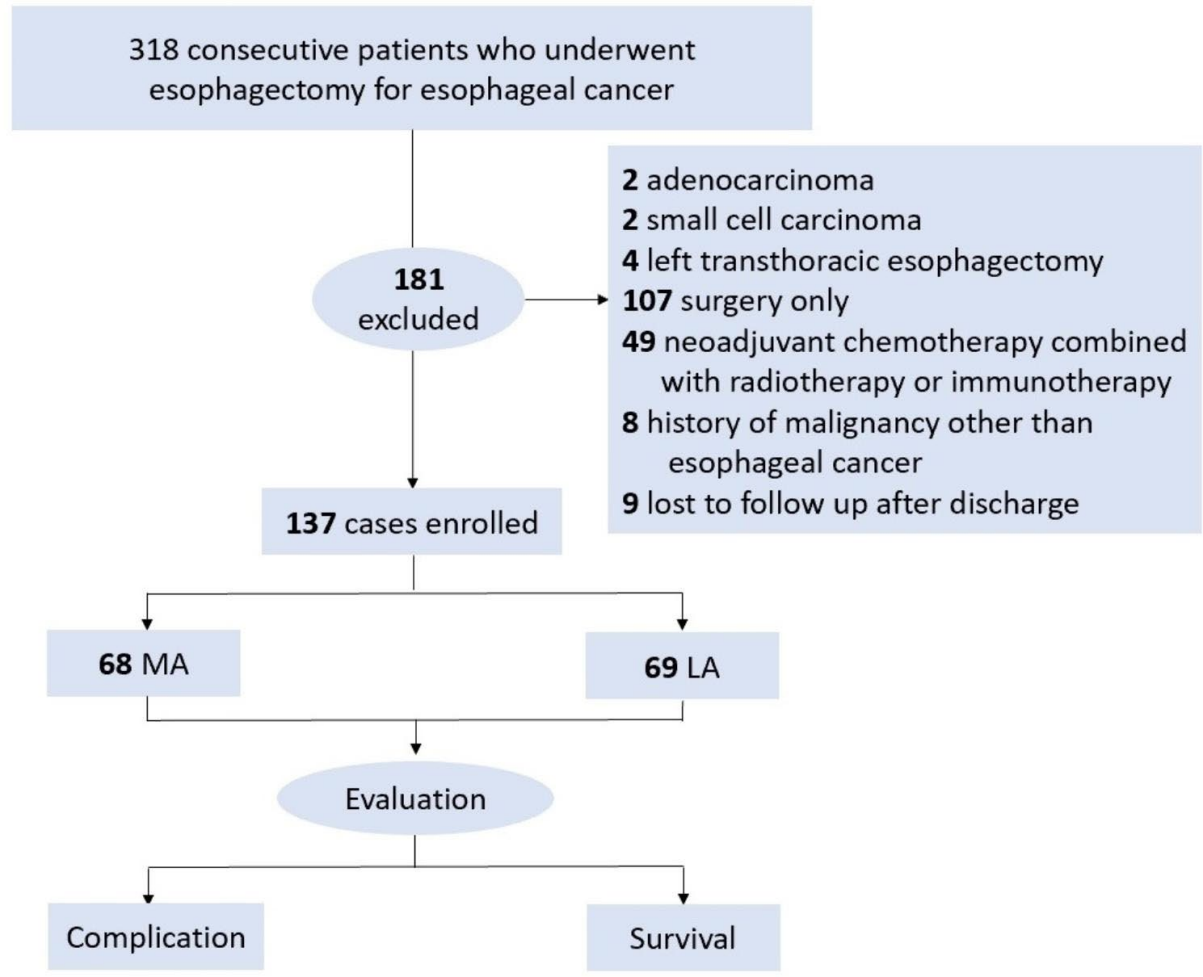
Study flow chart. AL, anastomotic leakage; MA, more decreased albumin; LA, less decreased albumin

**Fig. 2:**
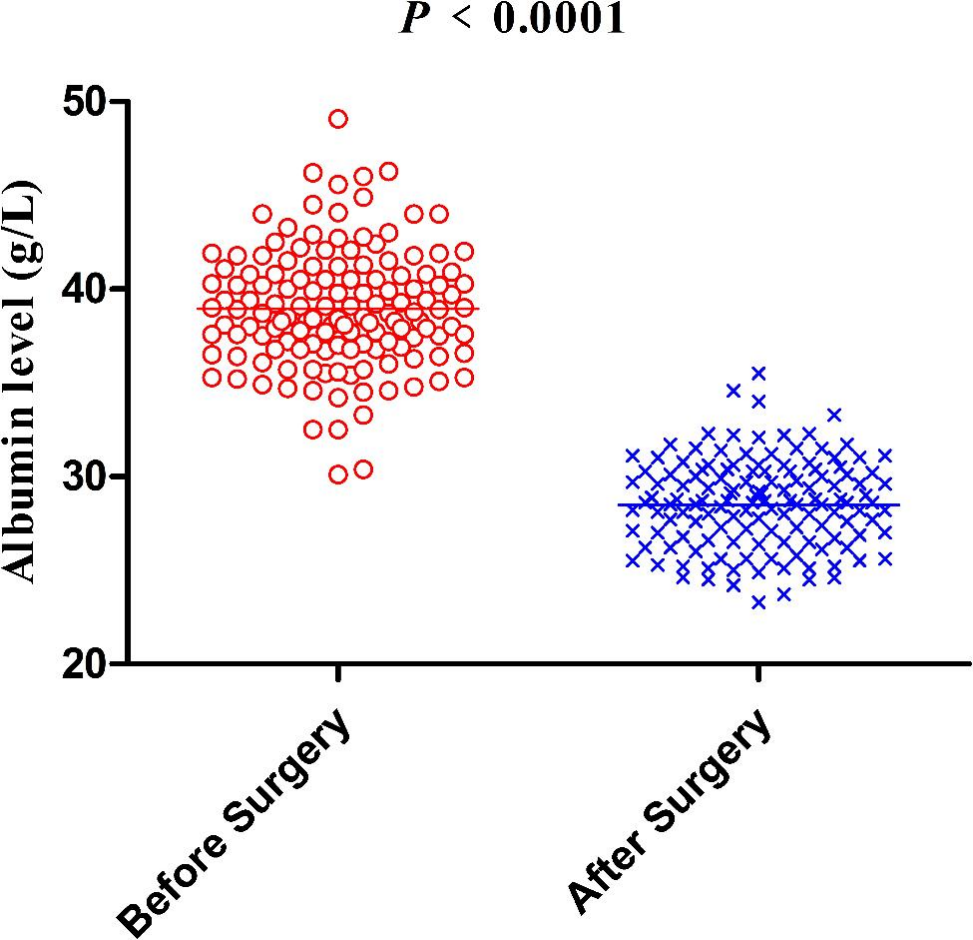
Preoperative serum albumin level

After the exclusion of 181 patients according to the exclusion criteria, the remaining 137 patients were retrospectively enrolled (Fig. 1). A total of 109 male and 28 female patients were included, with a median age of 65 (range: 47–85) years. After detecting the postoperative serum albumin in all patients, a significant decrease was observed comparing to the preoperative values (*p* < 0.0001) (Fig. 2). The average decreasing level of serum albumin was 10.4 ± 3.3 g/L (range from 1.0 to 18.4 g/L). Thus, the baseline of the average decreasing level (percentage) for serum albumin was determined as 26.3%. Sixty-eight patients were assigned to group MA, and 69 patients were assigned to group LA. There was no significant difference in the clinicopathologic characteristics, such as BMI (*p* = 0.120), tumor location (*p* = 0.658), clinical T stage (*p* = 0.281), clinical N stage (*p* = 0.821) and WHO performance status score (*p* = 0.851) between the two groups (Table 1).

**Table 1:** Baseline characteristics of patients*

Characteristic	MA (N = 68)	LA (N = 69)	*p* value
Age — yr			
Median	65	65	0.734
Range	47–85	50–80	
Sex — no. (%)			
Male	55 (80.9)	54 (78.2)	0.833
Female	13 (19.1)	15 (21.7)	
BMI — kg/m2	22.8	23.5	0.120
Tumor location — no. (%)			0.658
Proximal third	19 (27.9)	15 (21.7)	
Middle third	31 (45.5)	36 (52.1)	
Distal third	18 (26.4)	18 (26.1)	
Clinical T stage — no. (%)			0.281
cT1	0	0	
cT2	8 (11.7)	6 (8.6)	
cT3	50 (73.5)	58 (84.1)	
cT4	10 (14.7)	5 (7.2)	
Clinical N stage — no. (%)			0.821
N0	11 (16.1)	14 (20.2)	
N1	38 (55.8)	40 (57.9)	
N2	12 (17.6)	10 (14.4)	
N3	7 (10.2)	5 (7.2)	
WHO performance status score — no. (%)‖			0.851
0	49 (72.1)	48 (69.6)	
1	19 (27.9)	21 (30.4)	

MA, more decreased of serum albumin level; LA, less decreased of serum albumin level; BMI, body mass index; WHO, World Health Organization

* Percentages may not add up to 100 because of rounding

‖ WHO performance status scores are on a scale of 0 to 5, with lower numbers indicating better performance status; 0 indicates fully active, and 1 unable to carry out heavy physical work

When comparing the surgical outcome, none of the patients required intraoperative blood transfusion and emergency conversion to an open surgery. Similarly, there was no significant difference in the operation time (MA: 198.7 ± 36.4 min vs. LA: 195.7 ± 27.5 min, *p* = 0.585) and blood loss (MA: 44 ± 38.4 ml vs. LA: 38.3 ± 34.2 ml, *p* = 0.366). Five (5/68, 7.3%) patients received three-field lymphadenectomy due to the enlarged with suspicious metastasis lymph nodes were detected preoperatively in MA group while the same procedure of lymphadenectomy was performed in 7 (7/69, 10.1%) patients in LA group (*p* = 0.764). The number of total lymph nodes retrieved in MA group was 32.3 ± 12.9 and 33.2 ± 12.0 in LA groups (*p* = 0.650). Further to evaluate the number of stations of lymph node dissection, no statistically significant was found between groups (MA: 11.0 ± 1.7 vs. LA: 11.1 ± 1.6, *p* = 0.563) (Table 2).

**Table 2:** Surgical outcome

Characteristic	MA (N = 68)	LA (N = 69)	*p* value
Operation time - min	198.7 ± 36.4	195.7 ± 27.5	0.585
Blood loss - ml	44 ± 38.4	38.3 ± 34.2	0.366
LNs dissection - no. (%)			0.764
Two - field	63 (92.6)	62 (89.8)	
Three - field	5 (7.3)	7 (10.1)	
Retrieved LNs - no.			
Total	32.3 ± 12.9	33.2 ± 12.0	0.650
Station	11.0 ± 1.7	11.1 ± 1.6	0.563
Transfusion - no. (%)	0	0	
Conversion - no. (%)	0	0	
R0 Resection rate - no. (%)	66 (97.0)	68 (98.5)	0.620

MA, more decreased of serum albumin level; LA, less decreased of serum albumin level; LNs, lymph nodes

Percentages may not add up to 100 because of rounding

The total incidence of postoperative complications was 51.4% and 42.0% in MA group and LA group, respectively (*p* = 0.306) (Table 3). Among the 137 patients, a total of 8 (5.8%) patients experienced postoperative AL, seven (7/68, 10.2%) in MA group and one (1/69,1.4%) in LA group (*p* = 0.033). Two of the patients in MA group and one in LA group suffered a cervical AL without symptoms and managed by a nil-by-mouth regimen combined with enteral nutritional support. An intrathoracic AL was developed in the other 5 patients in MA group. Two of them with minimally symptomatic leakage were managed by fluid drainage and nutritional administrated. Unfortunately, the other three were dead even after endoscopic or surgical treatment. The mortality rate in MA group was 4.4% (3/68), a little higher than the overall setting of our database (1.8%), but no significant difference when compared with that in LA group (*p* = 0.120). When considering other common postoperative complications, no significant difference was exhibited between groups, such as arrhythmia (*p* = 0.745), pneumonia (*p* = 0.823) and incision infection (*p* = 0.202). When using the Clavein-Dindo system to classify the postoperative complications, the median grade of complications was II. Although more cases had a higher grade than IIIa complications in MA group, the difference was not statistically significance.

**Table 3:** Postoperative complications

Characteristic	MA (N = 68)	LA (N = 69)	*p* value
Complication - no. (%)			
Anastomotic leakage	7(10.2)	1(1.4)	0.033
Atelectasis	1(1.4)	2(2.8)	0.569
ARDS	2(2.9)	1(1.4)	0.620
Arrhythmia	4(5.8)	6(8.6)	0.745
Chylothorax	0	0	
Incision infection	4(5.8)	1(1.4)	0.202
Mediastinal abscess	3(4.4)	0	0.120
Pneumonia	11(16.1)	13(18.8)	0.823
Pneumothorax	2(2.9)	4(5.7)	0.681
Recurrent nerve paralysis	4(5.8)	6(8.6)	0.745
Thrombosis	0	1(1.4)	0.321
Hemorrhage	1(1.4)	0	0.433
Total	35(51.4)	29(42.0)	0.306
Clavien-Didone grade - no. (%)			
I	15(22.0)	18(26.0)	0.690
II	16(23.5)	13(18.8)	0.536
IIIa	2(2.9)	4(5.7)	0.681
IIIb	3(4.4)	0	0.120
IVa	2(2.9)	0	0.245
IVb	1(1.4)	0	0.496
V	3(4.4)	0	0.245
Median Clavien-Dindo	II	II	
Re-operation	1(1.4)	0	0.496
In hospital death	3(4.4)	0	0.120

MA, more decreased of serum albumin level; LA, less decreased of serum albumin level; ARDS, acute respiratory distress syndrome

NOTE: Data are presented as No. (%)

**Fig. 3:**
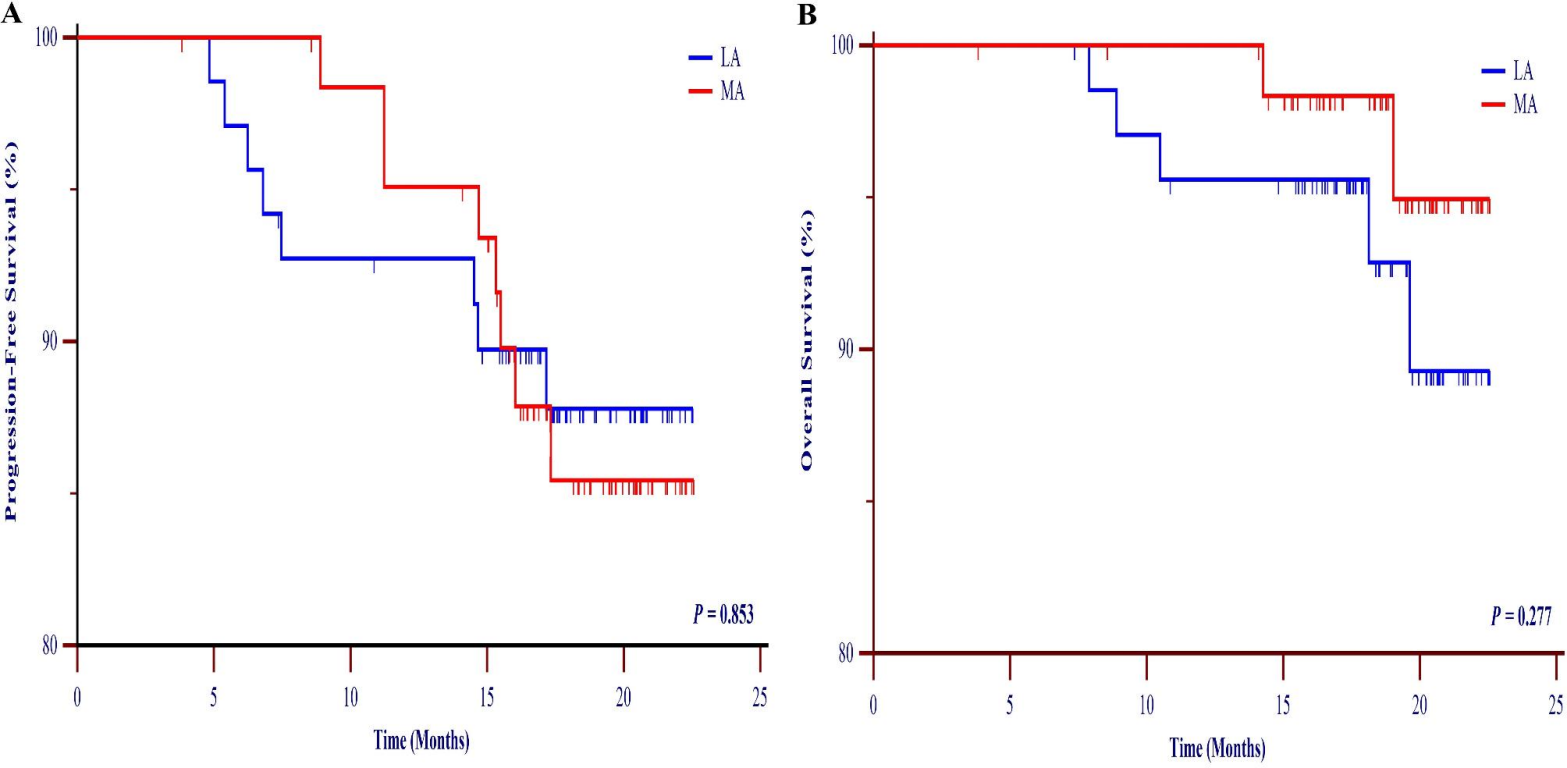
Progression-free survival (DSS) (**A**) and overall survival (OS) (**B**) curves of the MA group and LA group. AL, anastomotic leakage; MA, more decreased albumin; LA, less decreased albumin

Finally, the survival data of each group were compared to analyze the effect of decreased serum albumin levels on patient prognosis. A media period of follow-up is 22 months. PFS in LA group was a little higher than that in MA group, but it was no significant difference (*p* = 0.853) (Fig. 3A). Similarly, no difference was observed in OS between the two groups (*p* = 0.277) (Fig. 3B). These data indicated that patients who had less postoperative decreased serum albumin level did not have a survival benefit than those with more decreased levels.

## Discussion

AL is a severe complication following esophagectomy with high mortality. In spite of increasing research efforts and knowledge gain, pathophysiology and causal factors of AL remains unclear. Notably, the identification of risk factors for AL is pivotal for implementation of patient-tailored treatment regimen. For the intrinsic anatomic reason, lack of esophageal serosa is considered to contribute to the development of AL [12], meanwhile, malnutrition, neoadjuvant therapy and surgical technique may configure as extrinsic factors for the occurrence of AL [13]. Serum albumin is considered as one of the indicators of nutrition and associated with postoperative tissue healing has achieved wide acceptance [14]. In this study, the results suggested that the occurrence of AL is significantly higher in patients with greater perioperative decreased albumin level after treatment with NCT followed by MIE. However, the survival rate was not significant difference between groups, it is indicated that AL does not impair the survival benefit once recovered.

Several attempts had been made to establish a commonly accepted system to predict occurrence of AL, however, no consensus has been reached due to the broad and diversified criterion. As mentioned above, independently from intrinsic anatomic parameter, malnutrition, neoadjuvant therapy and surgical technique are imperative factors in the development of AL [13]. This relates as much to biology of the esophagus as to the surgical technique and perioperative management. To eliminate the heterogeneity of the participants, only ESCC patients treated with NCT followed by MIE were included in this study. Although MIE was demonstrated to be not associated with the occurrence of AL compared to open surgery [15], in this study MIE was performed by the experienced surgeons that can neglect the proficiency gain curve-associated morbidity [16]. Additionally, only patients treated with NCT were included in this study to keep homogeneity of the data.

More recently, management strategies have evolved from prioritizing the intervention after AL occurrence to primarily considering the prevention before AL development. Remarkable progression had been achieved on prediction of AL, e.g., intraoperative real-time monitoring of gastric conduit perfusion which is one of major causes for the development of AL [17, 18]. However, advanced medical devices required is limited implementation only in the high-volume center. Comparing with advanced technique-required methods, detection of serum albumin is a quick and simple way that is routinely handled in the clinical practice.

Hypoalbuminaemia, reflecting a poor nutritional status, which was associated with negative impact on the recovery and survival of patients in different clinical settings. Meanwhile, serum albumin was identified as an excellent parameter in the nutrition assessment and widely used in predicting the prognosis of patients, especially in the postoperative course of surgery. Previously, a certain value of serum albumin (e.g. 35 g/L) was identified as a cutoff for predicting AL after surgery in esophageal cancer patients [19, 20]. However, as a disease highly related to the dietary habits, chronic malnutrition with a low value of serum albumin can be observed in some of esophageal cancer patients, even before diagnosis. Likewise, neoadjuvant therapy is another cause that may lead patients into an unsteady physiological situation, resulting in a malnourished presentation. Furthermore, nutrition support was applied in some of patient with increasing serum albumin level intent preoperatively. However, the value of serum albumin cannot maintain for a long time due to the extrinsic supporting (the half-life of the albumin is about 20 days, and the changes in albumin can occur rapidly, especially in dysfunction of albumin metabolism). Finally, the value of serum albumin may be underestimated by saline infusion postoperatively [21]. For these reasons, serum albumin should be considered as a marker for predicting AL in a broad sense, rather than an instantaneous certain value. Hence, to address the question of whether an instantaneous certain value of serum albumin is accurate enough to predict AL, we investigate the relationship between AL and serum albumin by using the perioperative decreased level of it, which can be most likely to reflect the intrinsic physiological derangement of the patient.

Previous studies use less than 35 g/L, either preoperatively [19] or postoperatively [20], as a threshold value for the serum albumin level associated with the occurrence of AL was determined to provide guidance in the management of patients. However, in current study, nearly 10% (13/137, 9.5%) of patients receiving NCT had a preoperative serum albumin level of less than 35 g/l, meanwhile, almost all patients (135/137, 98.6%) with a lower level than 35 g/l postoperatively. Moreover, no significant difference was exhibited when 35 g/l of serum albumin level was defined as cutoff between groups, irrespective of performing surgery or not (preoperatively, 1/12 vs. 7/117, *p* = 0.556679; postoperatively, 1/1 vs. 7/128, *p* = 0.113783). To address the question of why the correlation disappeared in our study and whether this is a surrogate for the effect of neoadjuvant therapy, we chose to investigate this unusual phenomenon by using the decreased serum albumin level perioperatively as an object. Additionally, the serum level of albumin was dramatically decreased after MIE in patients receiving NCT, 35 g/l as a certain cutoff for the warning signal of the AL is not as sensitive as in patients treated with surgery alone. Fortunately, decreased level of serum albumin was identified as a significant predictive aspect for the occurrence of AL in current study. Nevertheless, we still believe that it is an issue of identifying a more sensitive and accurate threshold value of serum albumin other than 35 g/l in predisposing AL occurrence in patients receiving NCT.

In our previous study, the severely decreased serum albumin level after MIE was not associated with the occurrence of AL. The plausible reasons for the conflict results may be explained as follow: [1] First, the AL rate is less than half of incidence in current study (5.8%) comparing to our previous one (11.7%) [9]. This can most likely be explained by a remarkable progression of anastomotic technique that has been achieved in our center in past years, since surgical skill was regarded as a crucial aspect affecting the occurrence of AL [2]. Secondly, in contrast with our previous study, a small population-base study with 60 patients was conducted, more than twice number of participants has been enrolled in current study. This may contribute to obtaining more accurate results [3]. Third, all patients in current study were treated with NCT, which may have a certain impact on the nutritional status but the effect on the occurrence of AL is controversial, although radiation therapy is demonstrated to be associated with the increase in AL incidence [22]. Notwithstanding, the relationship between chemotherapy and AL rate is not established due to less consistent and unequivocal evidence had been emerged. Therefore, this is one of the reasons we chose to investigate this phenomenon using patients receiving NCT with a larger study population.

Although no significant difference was observed in postoperative complications between groups, three patients suffered perioperative death (3/68, 4.4%) in MA group, whereas no mortality was observed in LA group. All of the death was suffered from AL. One was caused by the severe infection resulting from AL. The other one died of hemorrhagic shock caused by a massive hemorrhage from the benches of aortic arch (may be left subclavian artery, because no autopsy, the position of the hemorrhage cannot be identified), which was the most severe complication of AL. The last one died of ARDS attributed to the result of AL. The main cause of death in patients with AL was the severe infection in this study, which was consistent with previous research [5]. Moreover, the common characteristic of the death was hypoalbuminemia, whose serum albumin was decreased at an extremely low level postoperatively. However, whether prophylactic intravenous infusion of albumin can reduce the incidence of AL is still lack of evidence. Before surgery, nutrition administration for patients receiving NCT may contribute to improving the tissue healing [23]. Once AL occurred, closure and coverage of leakage and fluid drainage are the principles of the management strategy.

Limited data had been provided to reveal the survival difference in patients with different serum albumin levels. In current study, survival benefit was not exhibited in patients with less perioperative decreased serum albumin level after a median period of 22 months follow-up. One of the reasons may be that serum albumin level in most patients would recover to normal status after discharge, regardless of decreased level. The hypoalbuminemia may influence the physiology of the patients only in an acute phase, after recovering, deleterious effects that impair clinical outcomes may disappear. Nevertheless, the follow-up is still ongoing, and whether survival difference existing remains need to be investigated.

Although knowledge of risk factors is increasing, the prioritization of them for the development of AL is not well defined. As a quick and simple approach, detection of serum albumin, may contribute to predicting the occurrence of AL. In this study, we demonstrated that perioperative decreased serum albumin level was associated with the occurrence of AL but did not impair the survival benefit in ESCC patients treated with NCT followed by MIE. However, limitations still exist in current study. First, the number of participants was not large enough. Second, this study was conducted retrospectively in a single center. Finally, to obtain the homogeneity of the data, only ESCC patients treated with NCT followed by MIE were enrolled. Whether current conclusion can be generalized remains unknown. Therefore, further study in wider populations is warranted to validate the efficacy of perioperative decreased serum albumin levels in esophageal cancer patients. Nevertheless, gaining insight into the efficacy of prevention strategies for AL is crucial. Meanwhile, from current study, the extremely decreased serum albumin level after MIE was indicative of a warning signal enabling surgeons to prevent the AL occurrence, which is a severe and potentially life-threatening complication after MIE.

## Data Availability

The datasets used and/or analyzed during the current study are available from the corresponding author upon reasonable request.

## Abbreviations

NCT: Neoadjuvant chemotherapy
pCR: Pathologic complete response
ESCC: Esophageal squamous cell carcinoma
MIE: Minimally invasive esophagectomy
MA: More decreased of serum albumin level
LA: Less decreased of serum albumin level AL:Anastomotic leakage
STROBE: Strengthening the reporting of observational studies in epidemiology
BMI: Body mass index
PFS: Progression-free survival
OS: Overall survival
AJCC: American Joint Committee on Cancer
UICC: Union for International Cancer Control
ARDS: Acute respiratory distress syndrome
LNs: Lymph nodes
WHO: World Health Organization
